# Correlation between white blood cell count at admission and mortality in COVID-19 patients: a retrospective study

**DOI:** 10.1186/s12879-021-06277-3

**Published:** 2021-06-14

**Authors:** Bin Zhu, Xiaokai Feng, Chunguo Jiang, Song Mi, Liya Yang, Zhigang Zhao, Yong Zhang, Liming Zhang

**Affiliations:** 1 Department of pharmacy, Beijing Tiantan Hospital, Capital Medical University, Beijing, 100160 China; 2 Department of Respiratory and Critical Care Medicine, Beijing Institute of Respiratory Medicine, Beijing Chaoyang Hospital, Capital Medical University, Beijing, 100020 China; 3 Cranio-maxillo-facial Surgery Department, Plastic Surgery Hospital of Peking Union Medical College, Chinese Academy of Medical Sciences, Beijing, 100144 China; 4 Department of Hepatobiliary Surgery, Union Hospital, Tongji Medical College, Huazhong University of Science and Technology, Wuhan, 430022 China

**Keywords:** Coronavirus disease-19, White blood cells, Death, Survival rate, Second quartile

## Abstract

**Background:**

Coronavirus disease-19 (COVID-19) has become a world health threaten. Its risk factors with death were still not known. White blood cells (WBC) count as a reflection of inflammation has played a vital role in COVID-19, however its level with death is not yet investigated.

**Methods:**

In this retrospective, single-center study, all confirmed patients with COVID-19 at West Branch of Union Hospital from Jan 29 to Feb 28, 2020 were collected and analyzed. Demographic and clinical data including laboratory examinations were analyzed and compared between recovery and death patients.

**Results:**

A total of 163 patients including 33 death cases were included in this study. Significant association was found between WBC count and death (HR = 1.14, 95%CI: 1.09–1.20, *p* < 0.001). The regression analysis results showed there was a significant association between WBC count and death (HR = 5.72, 95%CI: 2.21–14.82, p < 0.001) when use the second quartile as a cutoff value (> 6.16 × 10^9/L). The difference was still exist after adjusting for confounding factors (HR = 6.26, 95%CI: 1.72–22.77, *p* = 0.005). In addition, Kaplan-meier survival analysis showed that there was a significant decline of the cumulative survival rate (*p* < 0.001) in those with WBC count ≥6.16 × 10^9/L.

**Conclusion:**

WBC count at admission is significantly corelated with death in COVID-19 patients. Higher level of WBC count should be given more attention in the treatment of COVID-19.

## Background

Since December 2019, coronavirus disease 2019 (COVID-19) emerged and rapidly spread throughout world [1, 2]. The pathogen has been identified as a novel enveloped RNA beta coronavirus that has currently been named severe acute respiratory syndrome coronavirus 2 (SARS-CoV-2), which is sufficiently divergent from SARS-CoV [3, 4]. The most common symptoms are fever, dry cough, and fatigue. Ground-glass opacity (GGO), consolidation lesions, and reticular patterns were the common radiologic findings on chest computed tomography (CT) [5]. No antiviral treatment for coronavirus infection has been proven to be effective [1].

Patients with severe illness may progress to shortness of breath, and might develop acute respiratory distress syndrome (ARDS), septic shock, and require intensive care unit (ICU) admission [6, 7]. At this stage, the mortality rate is high. Older age with comorbidities, higher neutrophil-to-lymphocyte ratio, higher MuLBSTA score, higher Sequential Organ Failure Assessment (SOFA) score, and d-dimer greater than 1 μg/L on admission were associated with worse outcomes [7–9]. However, the data on the clinical characteristics at the early stage and outcomes of patients with SARS-CoV-2 infection remain scarce.

In this study, we investigated the white blood cells (WBC) count of patients with confirmed COVID-19 and a definite clinical outcome (death or discharge) who were admitted to the West Branch of Union Hospital in Wuhan. We aim to explore risk factors of severe disease and in-hospital death for patients, and help clinicians to identify patients on admission with poor prognosis.

## Patients and methods

### Patients

The COVID-19 patients’ clinical characteristics were retrospectively analyzed from January 29 to February 28, 2020 in the West Branch of Union Hospital in Wu Han province. All patients who were diagnosed with COVID-19 pneumonia according to WHO interim guidance were enrolled in the study [10]. Throat swab specimens were collected at admission, and the laboratory nucleic acid tests using real time polymerase chain reaction (RT-PCR) for COVID-19 RNA were conducted immediately in the Laboratory department of West Branch of Union hospital. Meantime, all patients recessive chest x-rays or chest CT to further identify the bilateral ground-glass opacity of infiltrates of lung. As the urgent situation on this emerging pathogen, the verbal consent was obtained before enrolment which was approved by the Ethics Committee of West Branch of Union hospital, Tongji Medical College, Huazhong University of Science and Technology. Confidential patient information was deleted from the entire data set prior to analysis.

### Laboratory assays

Fasting blood samples from the elbow veins of each participant were collected at admission. The hematological biochemical parameters comprising WBC count, neutrophil, serum lipid profiles and other index were examined in the Laboratory department of West Branch of Union hospital.

### Data collected

Medical records including death time and other clinical diagnosis and therapeutic schedules were carefully extracted using a standardized case report form. If information was not clear, then the doctors or other healthcare providers who were in charge were consulted.

### Statistical analysis

Data were presented as Means (SD) or medians (25th percentile-75th percentile) and proportions were calculated for population characteristics. Cox proportional hazard regression analysis was performed to evaluate the relationship between death and WBC count. In addition, we adjusted for age, sex, systolic blood pressure, diastolic blood pressure, body mass index, fasting glucose, total cholesterol, triglycerides and hdl cholesterol in the multivariable model. The relationship of death rate and WBC count was estimated using the Kaplan-Meier method. Survival differences between groups were compared using the log-rank test. All statistical tests were 2-sided with the significant level set at 0.05. Statistical analyzes were performed using Empower Stats (http://www.empowerstats.com) and the R software, version 3.3.1 (http://www.R-project.org/).

## Results

### Clinical characteristics of patients

A total of 163 patients (82 female) with an average 59.09 ± 14.01 were included in this study. Of them, 33 patients were dead at last. There were 71 females in non-death group and 11 in death group. The average age of non-death was 56.4 ± 13.5 years and the pneumonia severity index (PSI) was 50.6 ± 36.6. The average age of death was 70.3 ± 9.7 years and the PSI was 105.5 ± 22.2. Of the death cases, 19 patients reported with either hypertension, coronary heart disease or diabetes history and only14 patients were not reported with any of the mentioned disease history. The demographics characteristics are in Table 1.

**Table 1:** Baseline characteristics of the study participants by death

Variables	Stratified by Death		
	Non-Death	Death	*P value*
No.	130	33	
Female, n (%)	71 (52.3)	11 (33.3)	0.080
Age, y^a^	56.4 ± 13.5	70.3 ± 9.7	< 0.001
SBP, mmHg^a^	129.7 ± 16.9	132.0 ± 19.8	0.500
DBP, mmHg^a^	81.9 ± 10.6	78.1 ± 16.3	0.110
BMI, kg/m^2a^	23.9 ± 3.0	24.1 ± 3.8	0.828
PSI ^a^	50.6 ± 36.6	105.5 ± 22.2	< 0.001
WBC, ×10^9/L^a^	6.2 ± 3.3	10.3 ± 4.7	< 0.001
APO-A^a^	0.9 ± 0.3	0.7 ± 0.2	< 0.001
APO-B^a^	1.0 ± 0.2	1.0 ± 0.3	0.135
Fasting glucose, mmol/L^b^	5.8 (5.4, 6.9)	7.2 (6.1, 9.1)	< 0.001
Total cholesterol, mmol/L^b^	4.2 (3.7, 4.7)	3.9 (3.6, 4.3)	0.163
Triglycerides, mmol/L^b^	1.4 (1.0, 2.0)	1.3 (1.2, 2.0)	0.704
HDL cholesterol, mmol/L^b^	0.9 (0.8, 1.2)	0.8 (0.6, 1.0)	0.013
CURB.65			< 0.001
0	67 (51.5)	2 (6.1)	
1	42 (32.3)	4 (12.1)	
2	19 (14.6)	17 (51.5)	
3	2 (1.5)	10 (30.3)	

*BMI* Body mass index, *SBP* Systolic blood pressure, *DBP* Diastolic blood pressure, *HDL* High-density lipoprotein;

^a^For continuous variables, values are presented as mean ± SD

^b^Values are presented as median (IQR)

### Relationship of WBC and death

**Fig. 1:**
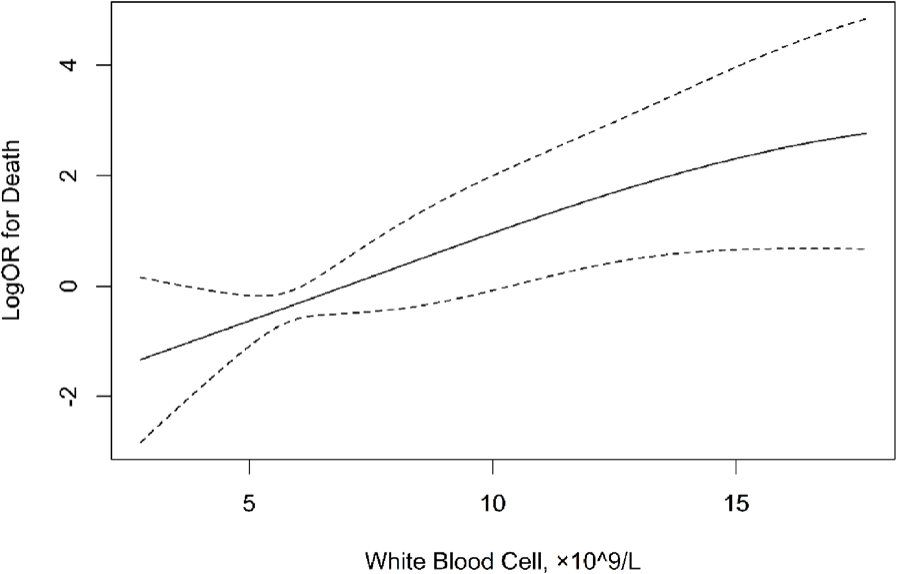
Smooth curves between WBC count and death

The relationship of WBC count and death are presented in Table 2. Significant associations were found between WBC count and death (HR = 1.14, 95%CI: 1.09–1.20, *p* < 0.001). Once adjust for covariables, the significance still exists (HR = 1.16, 95%CI: 1.07–1.25, *p* < 0.001). To further explore the influence of WBC count to death, we use second quartile and the normal range of the WBC count as a cutoff value to evaluate their relationship. The regression analysis results showed there was significant association between WBC count and death (HR = 5.72, 95%CI: 2.21–14.82, p < 0.001) when we use second quartile as a cutoff value (> 6.16 × 10^9/L). The significance was still existing even after adjusting for confounding factors (HR = 6.26, 95%CI: 1.72–22.77, *p* = 0.005); In addition, there was significant association between WBC count and death when the WBC count was use normal range as a cutoff value (HR = 3.76, 95%CI:1.82–7.77, *p* = 0.001), however, no significant difference was observed after adjusting for the confounding factors (HR = 2.08, 95%CI: 0.83–5.21, *p* = 0.118) (Table 2, Fig. 1).

**Table 2:** The association between fasting blood glucose and death

White Blood Cell, ×10^9/L	N	Case (%)	Crude model		Adjusted model^a^	*P value*
			HR (95%CI)	*P value*	HR (95%CI)	
WBC, as continuous	163	33 (20.2)	1.14 (1.09,1.20)	< 0.001	1.16 (1.07,1.25)	< 0.001
Categories1						
B1(< 6.16)	81	5 (6.2)	*Ref*		*Ref*	
B2(≥6.16)	82	28 (34.1)	5.72 (2.21,14.82)	< 0.001	6.26 (1.72,22.77)	0.005
Categories2						
< 10	141	22 (15.6)	*Ref*		*Ref*	
≥ 10	22	11 (50)	3.76 (1.82,7.77)	< 0.001	2.08 (0.83,5.21)	0.118

^a^Adjusted for age, sex, systolic blood pressure, diastolic blood pressure, body mass index, fasting glucose, total cholesterol, triglycerides, hdl cholesterol

**Fig. 2:**
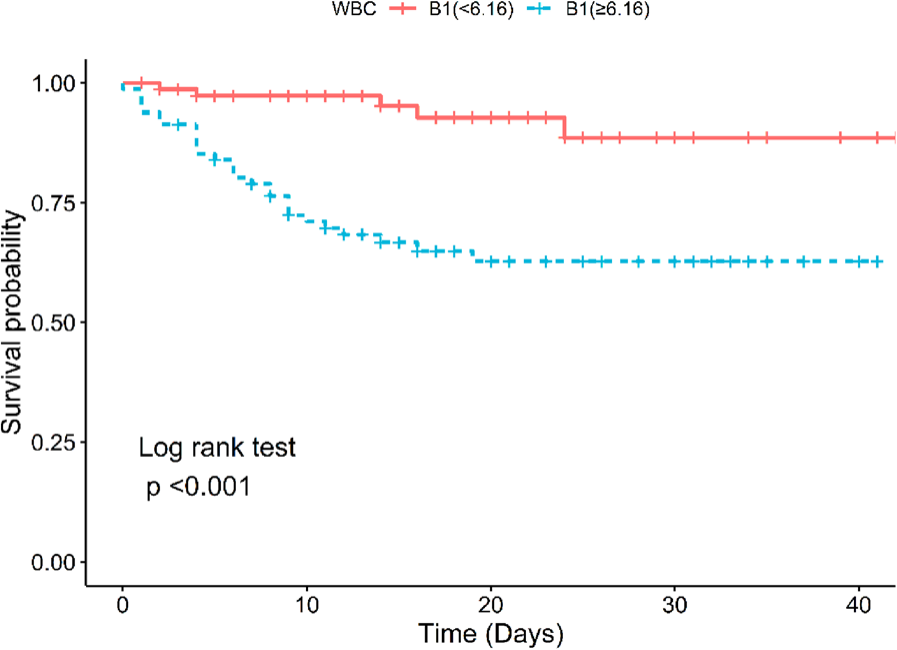
The survival rate of patients with COVID-19 which use second quartile of WBC count during hospitalization

In addition, Kaplan-meier survival analysis was also used to compare the variation trend of survival rate between the WBC ≥ 6.16 × 10^9/L and WBC<6.16 × 10^9/L during hospitalization. The results showed that there was a significant decline of the cumulative survival rate (*p* < 0.001) in those with WBC count ≥6.16 × 10^9/L (Fig. 2).

## Discussion

Nowadays, newly evolved coronaviruses have posed a global threaten to public health [11, 12]. Although, the epidemiological and clinical characteristics of patients were well documented, understanding of the clinical spectrum of COVID-19 infection is still limited. As a human-to-human transmission disease, middle-aged and elderly patients with underlying comorbidities are susceptible to respiratory failure and may have a poorer prognosis [13, 14]. Explore the risk factors related to the prognosis would be helpful for doctors to take an even more effective treatment. In this study, we systematically investigated the effect of WBC count on mortality. Our results showed that the death risk was associated with the WBC count at admission, although the index was at the normal range, those with higher WBC count patients were facing a much higher death possibility. This result was not reported elsewhere.

Although epidemiology and the genome had been well elucidated, much remain unknown. The risk factors which influence death are still not clear until now. The immune system is essential to control and eliminate CoVs infections. Nevertheless, accumulating evidence suggests that patients with severe COVID-19 might have a cytokine storm syndrome [15–17]. Patients of COVID-19 with maladjusted immune responses, may result in immunopathology and dead. Followed a deeper understanding of the interaction between coronaviruses and the innate immune systems of the hosts may shed light on the development and persistence of inflammation in the breath disease. Liu et al. had observed that nearly 80% of the patients had normal or decreased white blood cell counts, and 72.3% (99/137) had lymphocytopenia [18]. Zhang et al. had also reported a result of 9 patients, which their peripheral white blood cell counts were most normal and PCT were all negative [19]. These results were similar with ours. In our study we had found that most of the patients were with a normal range of WBC count. However, those with higher WBC lever patients were at a high risk of death.

Notable achievements have been made in understanding of COVID-19. As a spherical or pleomorphic enveloped particles, COVID-19 is the largest known viral with the size ranging from 26 to 32 kilobases [20]. However, the relationship of the virus with immune system is still unknow. Studies had reported significant increase of white blood cell and neutrophil, decrease of lymphocyte in severe patients [21, 22]. At present, there is no specific drug treatment against the new coronavirus in COVID-19 patients. The principles of treatment include control the symptoms and underlying diseases, active prevention of potential complications and secondary infections. Our results of WBC and death may shed light on the treatment of lung inflammation caused by CoVs.

Although our results might be helpful in COVID-19 treatment, the results should be considered as preliminary and some limitations could not be ignored. First, due to the limited number of patients, our conclusions need to be further verified by larger samples and multi-center data. Secondly, we had only observed the WBC count at admission, and a dynamic WBC count during the treatment were not observed. Thirdly, Our study had only observed a phenomenon, the potential mechanism still not known.

## Conclusion

In conclusion, our study suggests that WBC count at admission is significantly corelated with death in COVID-19 patients. Higher level of WBC count (≥ 6.16 × 10^9/L) should be given more attention in the treatment of COVID-19.

## Data Availability

All data analyzed during this study are included in this published article. The raw datasets used for the analysis are available from the corresponding author on reasonable request.

## Abbreviations

COVID-19: Coronavirus disease-19
WBC: White blood cells
SARS-CoV-2: Severe acute respiratory syndrome coronavirus 2
GGO: Ground-glass opacity
CT: Computed tomography
ARDS: Acute respiratory distress syndrome
ICU: Intensive care unit
SOFA: Sequential Organ Failure Assessment
2019-nCoV: 2019-novel coronavirus
RT-PCR: Real time polymerase chain reaction
PSI: Pneumonia severity index
